# Interaction of Various Variants of the Nanostructured Surface of Titanium with MSCs Isolated from Adipose Tissue

**DOI:** 10.3390/biomimetics6040061

**Published:** 2021-10-18

**Authors:** Ekaterina A. Gosteva, Alexander B. Dymnikov, Vitaliy V. Starkov, Daria M. Sedlovets, Marat P. Valikhov, Dmytryi A. Vishnevsky, Vladimir P. Chekhonin, Gurgen A. Tumanyan, Masound K. Ahmad

**Affiliations:** 1 Department of the Material Science of Semiconductors and Dielectrics, National University of Science and Technology MISiS, 4 Leninskiy Prospekt, 119049 Moscow, Russia; 2 Academy of Engineering, RUDN University, 117198 Moscow, Russia; 3 Department of Maxillofacial Surgery and Surgical Dentistry of the FSAEI, The Peoples’ Friendship University of Russia, 6 Miklukho-Maklaya St., 117198 Moscow, Russia; al.dymnikov@gmail.com (A.B.D.); dr.gurgentumanyan@gmail.com (G.A.T.); masoodkhosrawi@mail.ru (M.K.A.); 4 Institute of Microelectronics Technology and High Purity Materials of the Russian Academy of Sciences, Academician Ossipyan Str., 142432 Chernogolovka, Russia; starka@iptm.ru (V.V.S.); sedlovets@iptm.ru (D.M.S.); 5 Department of Basic and Applied Neurobiology, V. Serbsky National Medical Research Center for Psychiatry and Narcology, Kropotkinsky Lane 23, 119034 Moscow, Russia; marat.valikhov@gmail.com (M.P.V.); logannaridenna@gmail.com (D.A.V.); chekhoninnew@yandex.ru (V.P.C.)

**Keywords:** biocompatibility, chemical etching, denture, graphene-like films, mesenchymal stem cells, osseointegration, oxidation, passivation, surface nanostructuring

## Abstract

Titanium has been successfully used in dental implantology for a long time. Due to the osseointegration process, titanium implants are able to withstand the chewing load. This article is devoted to the study of surface treatment methods of titanium alloys and the study of their interaction with mesenchymal stem cells (MSCs). The surface microrelief can influence MSC differentiation in different ways, which subsequently gives it osteogenic potential. The paper proposes modes of surface modification of titanium alloys on Grade 4 and Grade 1 by chemical and electrochemical (anodizing) etching. The possibility of modifying the surface of titanium alloys using the synthesis of graphene layers has been proposed in this paper for the first time. The osteogenic potential of a particular surface was assessed by the number of mesenchymal stem cells cultured on them under identical conditions.

## 1. Introduction

The study and the application of cellular technologies for repairing bone defects is a multidisciplinary task that does not merely require research in the field of directed differentiation and cell growth, but also the search for optimal materials to create a solid framework for bioengineering design [1,2,3,4,5].

In contemporary maxillofacial surgery and surgical dentistry, the restoration of defects and deformities of bones is an urgent issue. Over the past 50 years, the view on prosthetics and restoration of the functions of the “chewing organ” has changed dramatically. In connection with the development of dental implantation, the direction and focus of research have also changed dramatically. During the existence of dental implantation as a direction in dental surgery and orthopedics, the experience has been accumulated and the basic ideas about the nature of the interaction of implanting material with bone have been formed [6].

Since the beginning of the history of dental implantation, titanium has been the optimal implantological material. Titanium has several advantages, such as high biocompatibility, bioinertness, good corrosion resistance due to the formation of a passivating oxide layer on the surface, non-magnetic properties, low thermal conductivity, and low coefficient of linear expansion; it is also virtually not toxic and has a relatively lower specific gravity than steel. Titanium is also distinguished by the constancy of physico-chemical properties in a wide temperature range.

It is well known that titanium is useful as a substrate for cell culture. The biocompatibility of titanium implants has been tested on various cells, for example fibroblasts, osteoblasts, chondrocytes, and bone marrow stromal cells [7,8,9,10]. To improve the interaction of cells with titanium implants, their surface has been modified in various ways [11].

To achieve good osseointegration, recruitment of osteogenic cells, such as osteoblasts and MSCs, is necessary. Cell adhesion is the first stage of interaction between cells and implants after biomaterial implantation, which consists of four stages: protein adsorption, cell contact with the material, attachment, and spreading [12,13]. It was proved that the quality of adhesion is crucial for modulating the ability of cells to proliferate and differentiate [14,15,16,17]. It was also shown that the differentiation of MSCs may depend on the surface of titanium.

This paper presents the results of nanostructuring of the surface of dental implants based on titanium alloys Grade 4 and Grade 1 by chemical etching and anodizing [18]. Chemical etching of the surface of the samples was carried out in solutions of various ingredient compositions and various temperature conditions. The possibilities for surface modification are significantly expanded by using various modes of anodizing the surface of the sample. For the first time in this study, it is also proposed to modify the surface properties of titanium implants by forming nanostructured graphene-like layers on their surface. The synthesis of graphene-like coatings was carried out by the CVD method (chemical vapor deposition) [19].

MSCs obtained from adipose tissue were grown on the surface of all samples under identical conditions [20]. After a three-day cultivation on titanium surfaces, MSCs were counted on the same area of samples. The experimental results allowed us to determine the optimal modes of chemical and electrochemical modification of the titanium surface, as well as the preferred synthesis modes of graphene layers on the titanium surface for a more favorable MSC growth.

## 2. Materials and Methods

### 2.1. Materials and Reagents

Grade 4 titanium alloys were used to conduct experiments on the formation of the developed surface and to study the process of osseointegration. Grade 1 titanium alloys were also used to evaluate and compare the results. Grade 1 titanium, which is also used to create implants, is inferior in its mechanical properties than Grade 4. Chemical composition and the mechanical characteristics of these alloys are shown in Table 1 and Table 2.

The formation of a nanostructured surface of titanium alloys of Grade 4 and Grade 1 was carried out by chemical and electrochemical etching. The effectiveness of this method of forming a developed surface has been repeatedly covered in the literature [21,22,23,24].

Chemical etching was carried out in electrolytes based on hydrofluoric acid (HF) and ethyl alcohol (C_2_H_5_OH) in a 1:2 ratio of components. The etching was carried out at room temperature and normal pressure, and the etching time varied from 1 to 10 min. The best results were obtained by holding the samples in the solution for 5 min.

To determine the effect of the electrolyte composition on the morphology of the formed surface, etching was carried out in a solution based on dimethylformamide (DMF) with hydrofluoric acid (HF) in a ratio of 10:1 at a temperature of 20 °C, as well as etching in a boiling aqueous solution of hydrochloric (HCl) and sulfuric (H_2_SO_4_) acids in a ratio of 1:1 at a temperature of 110 °C.

The formation of a continuous homogeneous coating of titanium dioxide can perform a protective function, prevent corrosion, and introduce alloy components into the surrounding nutrient medium; it also improves the osseointegration properties of implants, which was shown in [25,26,27] when the anodizing of chemically treated samples was carried out. The process was carried out in the galvanostatic mode at a temperature of 20 °C (current parameters j = 35, 70, 105 mA/cm^2^). The process time ranged from 1 s to 10 min. It was found that the thickness of the formed oxide coating is directly dependent on the time of the anodizing process.

For the first time in this work, the formation of a protective coating based on graphene-like layers was proposed. There are references in the literature that graphene and graphene-like structures have high biocompatibility, for examplein [28,29,30,31]. Research in the biomedical applications of graphene is expanding, but, to date, it is mostly in its infancy.

The formation of graphene-like structures was carried out by ethanol vapor pyrolysis using standard gas-phase chemical synthesis (CVD) technology [19]. The process was carried out under reduced pressure (1 kPa) in a stream of an inert carrier gas (2 L/h of argon 99.9999%). A water-–alcohol mixture (distilled water and ethanol in various ratios) was injected directly into the reactor gas line using a peristaltic pump. The deposition temperature was 950 °C with a synthesis time from 35 to 120 min.

### 2.2. Research Equipment

Raster images of porous structures were obtained for determining the morphology and pore size of the obtained structure using a JSM-6700F field emission scanning electron microscope with an attachment of a Jeol 2300F energy dispersive microanalyzer.

To determine the roughness of the relief of the samples under study, we used the Alpha-Step IQ Surfaceprofiler ASIQ setup from KLA-Tencor (USA), which makes it possible to measure micro roughness with a resolution of up to 0.1 nm both at short scanning distances and for scans up to 10 mm in length. Computer control of the device and analysis and processing of the obtained data allow the reduction of the influence of factors not of plane parallelism and the surface bending of the samples.

Cell visualization was performed using a Nikon A1R MP confocal laser scanning microscope using a Nikon Plan Apo 20x/0.75 DIC N2 ∞/0.17 WD 1.0 objective. For visualization, plastic cups with a bottom composed of a 0.17 mm thick coverslip were used (SPL Lifesciences). Titanium discs were transferred to plates in a phosphate buffer solution. To fully visualize the surface of the titanium disk, a panoramic survey was carried out, consisting of a series of pictures of the field of view with a resolution of each frame equal to 1024 × 1024 pixels, after which the image was stitched into a single panorama.

Images were processed using NIS-Elements (Nikon) software. The number of cells on a titanium disk was counted due to binarization of visualized nuclei by the method of semi-automatic selection of image pixel intensity boundaries, and a further automatic calculation of the number of objects is defined in the image. Images were preliminarily processed using noise reduction methods (Bayse and Original Advanced Denoising) and image background reduction (Rolling Ball Correction). In the beginning, the raw image was processed using the noise reduction algorithm—Bayse Advanced Denoising, after which the original Advanced Denoising algorithm was applied. The last step was to remove the background image using the Rolling Ball Correction function. After processing, binarization and cell counting were performed on the image.

### 2.3. Obtaining the Mesenchymal Stem Cells

Mesenchymal stem cells were obtained from the adipose tissue of experimental Wistar rats according to the protocol [16], which were subsequently planted on titanium surfaces placed on the bottom of a 48-well plate. Cells were cultured for 3 days on DMEM/F12 medium (Gibco) with a 10% fetal bovine serum (Hyclone) and an antibiotic-antimycotic solution (Gibco). Next, the cells were fixed with a 4% formaldehyde solution and stained with nuclear dye DAPI.

## 3. Results and Discussion

### 3.1. The Choice of Material Samples for the Experiment

For the production of dental implants, technically pure titanium of 4 grades (Grade 1–4 ASTM, ISO) and titanium alloy Ti-6AI-4V (ASTM, ISO) are used. The most durable of the materials under consideration is the Ti-6AI-4V alloy. However, studies in 1984 showed that the vanadium contained in it should cause concern, since this metal has a toxic effect on biological subjects [7]. In addition, the degree of tissue adhesion that is made of titanium alloys is somewhat worse than to unalloyed titanium [8]. To date, it is universally recognized that the presence of toxic elements in the cultivated materials is unacceptable. Thus, from the point of view of better biocompatibility, substances belonging to the group of “pure” titanium appear to be more promising. It should be noted that, when it comes to “pure” titanium, only one of the 4 grades of titanium (Grades 1–4) is approved for introduction into the tissues of the body, in accordance with international standards.

The four grades of titanium are different in chemical composition, which, in fact, determines the biological compatibility and mechanical properties. Moreover, the strength of these materials is important. The best characteristics in this regard are those of titanium Grade 4. The chemical composition and mechanical properties of these alloys are shown in Table 1 and Table 2.

Titanium is widely used not only in surgical dentistry and maxillofacial surgery, but also in other areas of surgery and implantology [5,6,7]. A large number of works [8,9,10] are devoted to the study of titanium surface treatment methods.

Currently, studies are underway on the interaction of mesenchymal stem cells (MSCs) with various types of titanium surfaces [11]. Good results of interaction with stem cells are shown by the nanostructured titanium surface [12]. Studies are being conducted on the effect of the galvanic effect of sprayed Si/CaCO_3_ on the titanium surface on the osteogenic differentiation of MSCs [13]. This issue needs to be studied, since the surface of the osteogenic potential will significantly improve the existing technologies in dental implantation and bone grafting.

In our work, we used “pure” Grade 4 titanium, according to the ASTM standard, as:

(a) This material does not contain toxic vanadium, such as, for example, Ti-6AI-4V alloy;
(b) The presence of Fe in its composition (measured in tenths of a percent) cannot be considered negative, since, even in the event of the possible release of iron ions into the surrounding tissues, their effect on the tissues is not toxic, as in vanadium;
(c) Titanium Grade 4 has better strength properties compared to other materials of the “pure” titanium group.

### 3.2. Chemical and Electrochemical Surface Modification

**Figure 1:**
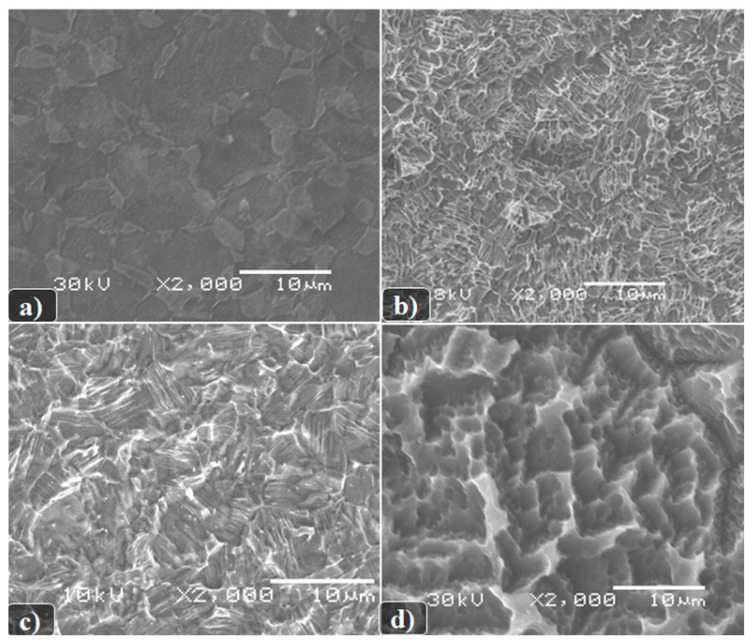
SEM image of the surface of the studied samples of titanium disks after: (**a**) Chemical etching in a DMF + HF. t = 1 min, T = 20 °C (material Grade 4); (**b**) Boiling in HCl solutions: H_2_SO_4_ (1:1), t = 9 min, T = 110 °C (material Grade 4); (**c**) Chemical etching in a solution of DMF + HF (1:10), t = 1 min, T = 20 °C (material Grade 1); and (**d**) Boiling in HCl solutions: H_2_SO_4_ (1:1), t = 9 min, T = 110 °C (material Grade 1).

As it can be seen from the presented images in Figure 1, the formed surface morphology depends both on the etching conditions themselves and on the composition of the titanium alloy used. In this case, the following dependencies can be observed:

- A more developed surface is formed as a result of chemical etching in boiling solutions of a mixture of sulfuric and hydrochloric acids;
- The etching of Grade 4 samples is slower than Grade 1, which is consistent with the published data and depends on the chemical composition of the alloy;
- We can see that the chemical etching of Grade 4 begins mainly at the grain boundaries and on surface defects.

**Figure 2:**
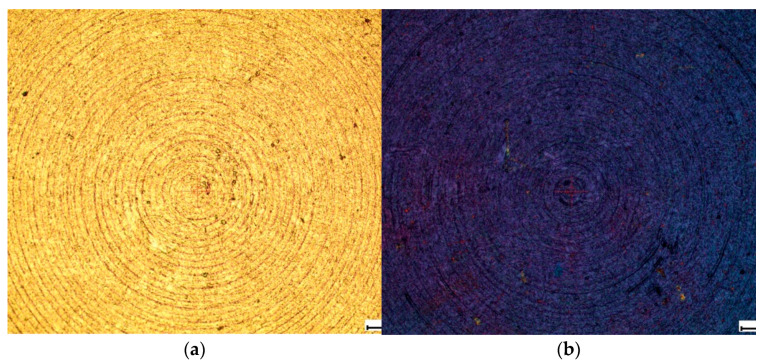
Image of the surface of the studied samples in an optical microscope: (**a**) without TiO_2_ coating and (**b**) with TiO_2_ coating.

Some samples after chemical etching were additionally anodized in water. The process was carried out in a galvanostatic mode under standard conditions. As a result of the electrochemical treatment, a continuous coating of titanium dioxide was formed on the surface of titanium alloys, the thickness of which was controlled by the time of the electrochemical treatment and could be estimated by the tint color shown in the Figure 2.

However, the study of samples using SEM was not informative, since the surface layer of TiO_2_ in the raster images is transparent and does not make noticeable changes. It is possible to evaluate the effect of the coating on the morphology of the obtained structure by measuring surface roughness using an Alpha-Step IQ Surfaceprofiler ASIQ profilometer.

To determine the roughness of the relief, measurements were carried out at five different points on the surface of the studied samples. The surface roughness was estimated by the average roughness (Ra), in nm, and RMS roughness (Rq), in nm (measurement error is within 5 angstroms). The roughness development parameters are summarized in Table 3.

### 3.3. Graphene-Based Coating Formation

Graphene nanocrystallites on the surface of Grade 4 and Grade 1 were synthesized in the CVD process at a temperature of 950 °C by the method of pyrolysis of vapors of water–alcohol mixtures under reduced pressure.

**Figure 3:**
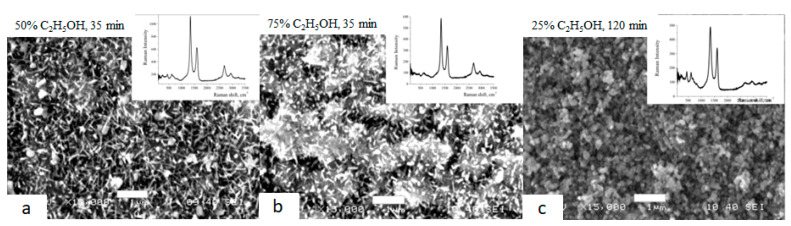
SEM images of the surface of Grade 4 titanium after graphene synthesis. In the upper right corner of each image are the corresponding Raman spectra.

The samples shown in Figure 3a,b were synthesized for 35 min at ethanol concentrations of 50% and 75%, respectively. The sample shown in Figure 3c was synthesized for 120 min at a concentration of 25%. Raman spectra have intense peaks characteristic of graphene nanocrystallites (D, G, 2D). The presence of TiO_2_ (bands up to 700 cm^−1^) in the form of nano needles (Figure 3a,b) and short rods (Figure 3c) is observed simultaneously.

### 3.4. Cultivation of Mesenchymal Stem Cells on Test Surfaces

**Figure 4:**
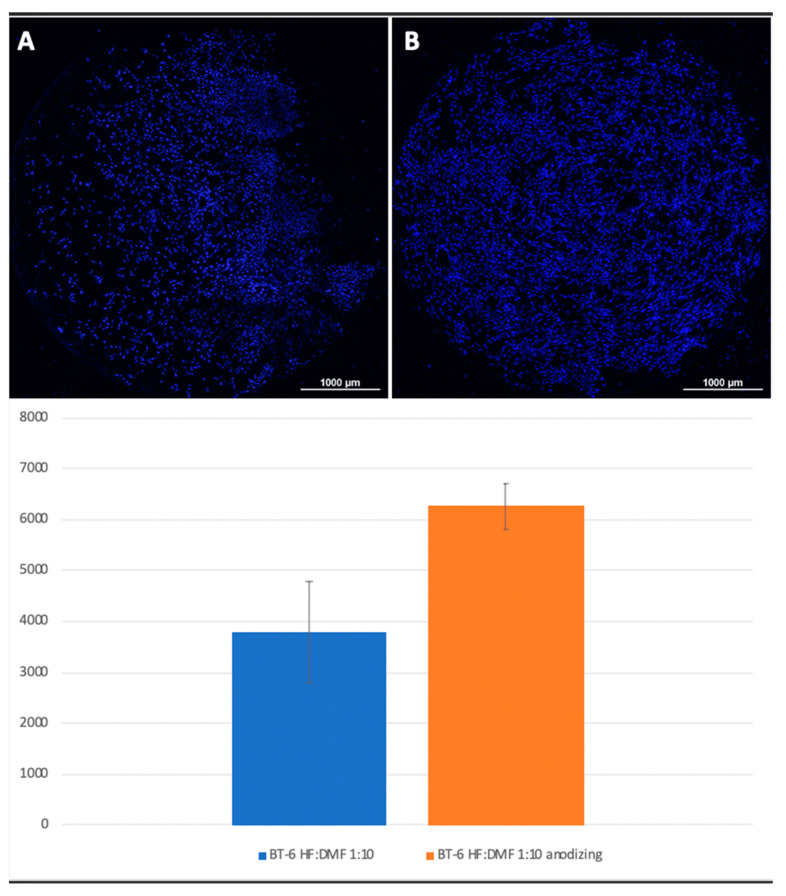
Comparison of the number of MSCs on the titanium surfaces of Grade 6 nanostructured with HF: DMF (**A**) and HF: DMF with anodization (**B**). Cell nuclei stained with DAPI stain.

**Figure 5:**
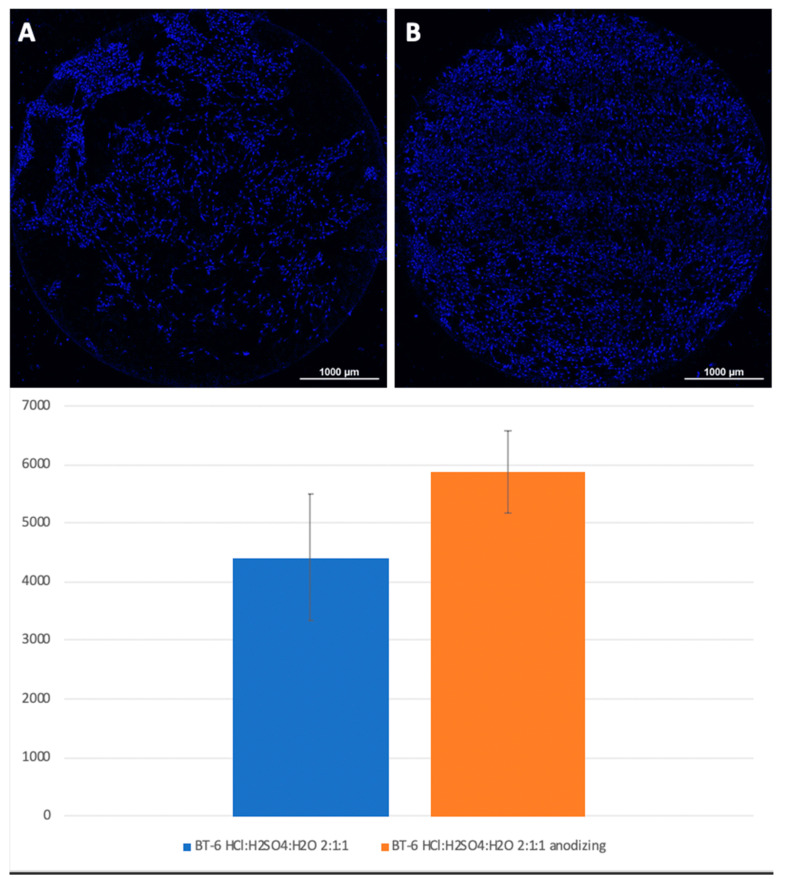
Comparison of the amount of MSCs on titanium surfaces nanostructured with HCl: H_2_SO_4_: H_2_O (**A**) and HCl: H_2_SO_4_: H_2_O with anodization (**B**). Cell nuclei stained with DAPI.

**Figure 6:**
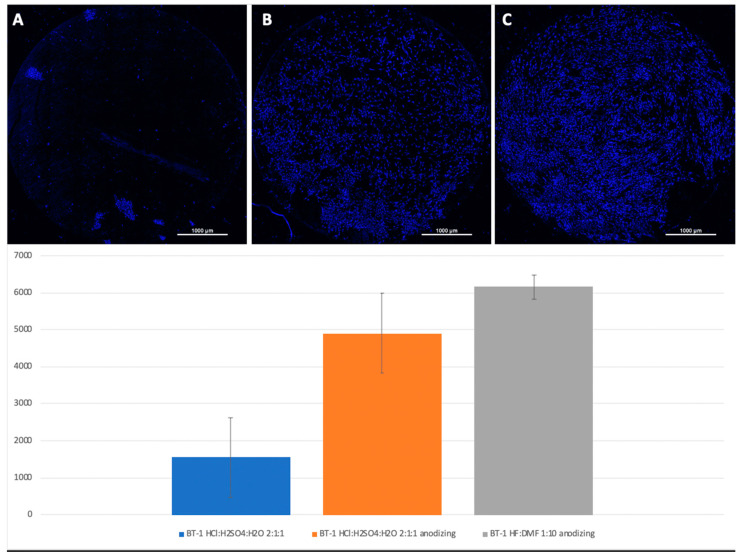
Comparison of the amount of MSCs on Grade 1 titanium surfaces nanostructured with HCl: H_2_SO4: H_2_O (**A**), HCl: H_2_SO_4_: H_2_O with anodization (**B**), and HF: DMF with anodization (**C**). Cell nuclei stained with DAPI.

Mesenchymal stem cells were planted on titanium surfaces of Grade 4 and Grade 1 nanostructured using HF: DMF (Figure 4) and HCl: H_2_SO_4_: H_2_O (Figure 5), with and without anodization. In the samples with anodization, the number of cells was higher. Similar results were obtained for titanium surfaces of Grade 1, nanostructured in the same way (Figure 6).

**Figure 7:**
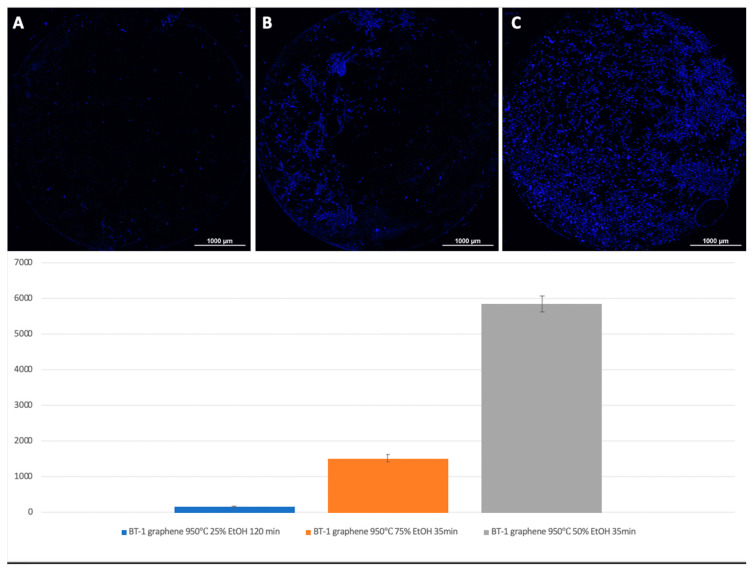
Comparison of the number of MSCs on titanium surfaces of Grade 1 coated with graphene 950 °C 25% EtOH 120 min (**A**), 950 °C 75% EtOH 35min (**B**), and Grade 1 graphene 950 °C 50% EtOH 35min (**C**). Cell nuclei stained with DAPI.

For Grade 1 titanium samples with graphene coating, the best results were obtained at a 50% ethanol content, a deposition temperature of 950 °C, and 35 min of synthesis (Figure 7).

**Figure 8:**
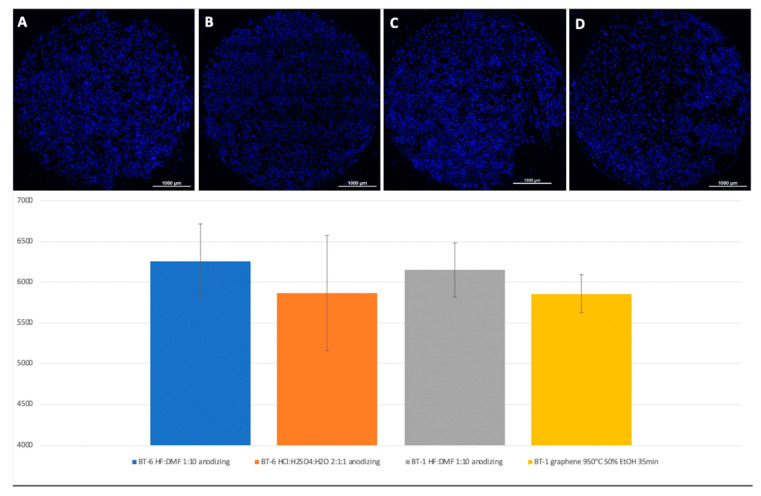
Comparison of the number of MSCs on the titanium surfaces of Grade 6, nanostructured with HF: DMF with anodizing (**A**), HCl: H_2_SO_4_: H_2_O with anodizing (**B**), Grade 1 HF: DMF with anodizing (**C**), and Grade 1 graphene 950 °C 50% EtOH 35min (**D**). Cell nuclei stained with DAPI stain.

A visual assessment of the distribution of cells on the surface of titanium disks allows us to conclude that, when the number is more than 5000 cells, the surface is filled uniformly and homogeneously. Best adhesion values were found in samples with nanostructured surfaces of HF: DMF with anodization—Grade 4 and Grade 1 (N = 6260 and 6151, respectively). Samples treated with HCl: H_2_SO_4_: H_2_O and anodizing also showed satisfactory adhesion (Grade 1–4902, Grade 4–5868). The best performance of samples coated with graphene film were found in samples Grade 1 graphene 950 °C 50% EtOH 35min (5855), which is also a satisfactory result (Figure 8).

## 4. Conclusions

The methods used to modify the surface of titanium-based alloys make it possible to change the surface roughness and morphological characteristics in a wide range of parameters. These capabilities will allow the successful use of a particular technique of nanostructuring the surface when creating prostheses based on titanium and its alloys.

Based on the results obtained, it is concluded that the most developed surface morphology is possessed by samples subjected to a combination of chemical etching and electrochemical etching with the following modes: Boiling in HCl: H_2_SO_4_ (1:1) solutions, t = 9 min, T = 110 °C + anodizing in H_2_O t = 1 s, T = 20 °C (material Grade 1).

To confirm the adhesive properties of the obtained samples, a mesenchymal stem cell culture was planted on them. It was found that cells interact better with surfaces that are nanostructured by anodizing. The best indicators of cell adhesion were revealed in samples for graphene coatings; the best results were shown for a 50% ethanol content, deposition temperature at 950 °C, and 35 min of synthesis.

The revealed number and nature of the distribution of cells on samples with anodization and coated with graphene at 50% ethanol content with a deposition temperature of 950 °C and 35 min of synthesis may indicate that the obtained surfaces are more favorable for cell adhesion, proliferation, and further differentiation, compared with other samples, which is consistent with data obtained by other research groups.

A comparative assessment of the adhesion properties of graphene coatings with samples of the anodized surface revealed similar cell adhesion indicators, which allows us to consider graphene coatings as a method for treating titanium surfaces to study their interaction with MSCs and impart an osteogenic potential to the surface.

Thus, the wide possibilities and effectiveness of using the above methods for nanomodification of the surface of prostheses based on titanium alloys for use in biomedical purposes are shown.

The studies performed in this paper are an important stage in the development of titanium implants for preclinical studies.

## Figures and Tables

**Table 1:** The chemical composition of titanium alloys used in medicine according to ISO 58321 II and ASTM F 67-89.

Element	Grade 1 (%)	Grade 2 (%)	Grade 3 (%)	Grade 4 (%)	Ti-6AI-4V Alloy (%)
**Nitrogen**	0 03	0 03	0 05	(0.05)	no
**Carbon**	0.1	0.1	0.1	0.1	(0.1)
**Hydrogen**	0.015	0.015	0.015	0.015	(0.015)
**Iron**	0.2	0.3	0.3	0.5	(0.4)
**Oxygen**	0.18	0.25	0.35	0.5 (0.4)	(0.2)
**Aluminum**	no	no	no	no	(5.5–6.75)
**Vanadium**	no	no	no	no	(3.5–4.5)
**Titanium**	Rest	Rest	Rest	Rest	Rest

**Table 2:** Mechanical properties of titanium alloys used in medicine according to ISO 58321-II and ASTM F 67-8.

	Grade 2 (MPa)	Grade 3 (MPa)	Grade 4 (MPa)	Ti-6AI-4V Alloy (MPa)
**Ultimate tensile strength**	240	345	450	550
**Yield strength**	170	230 (275)	300 (380)	440 (483)

**Table 3:** Roughness data of the studied samples.

Sample	Ra, nm	Rq nm
Grade 4, HF: DMF	134.6	176.4
Grade 4, HF: DMF + anod	150.2	199.6
Grade 4, boiling HCl: H_2_SO_4_	219	284.2
Grade 4, boiling HCl: H_2_SO_4_ + anod	238.6	298.8
Grade 1, HF: DMF	82.92	106.7
Grade 1, HF: DMF + anod	206.2	240
Grade 1, boiling HCl: H_2_SO_4_	185.2	228
Grade 1, boiling HCl: H_2_SO_4_ + anod	243.6	296.8
graphene 25% H_2_O, 35 min	216	253.8
graphene 50% H_2_O, 30 min	194.8	228.6
graphene 50% H_2_O, 35 min	240.2	280
graphene 75% H_2_O, 120 min	166.4	203.8
